# Supplementary material for: The interactome of intact mitochondria by cross-linking mass spectrometry provides evidence for coexisting respiratory supercomplexes
Source: Mol Cell Proteomics. 2017 Dec 8;17(2):216–32. doi: 10.1074/mcp.RA117.000470 (PMC5795388; doi:10.1074/mcp.RA117.000470)
Supplement: Supplemental Data [file supp_RA117.000470_133922_0_supp_23978_fzffwf.zip › spectra_annotation/mito_spectra_annotation/76-1-1-1-3-1.pdf]

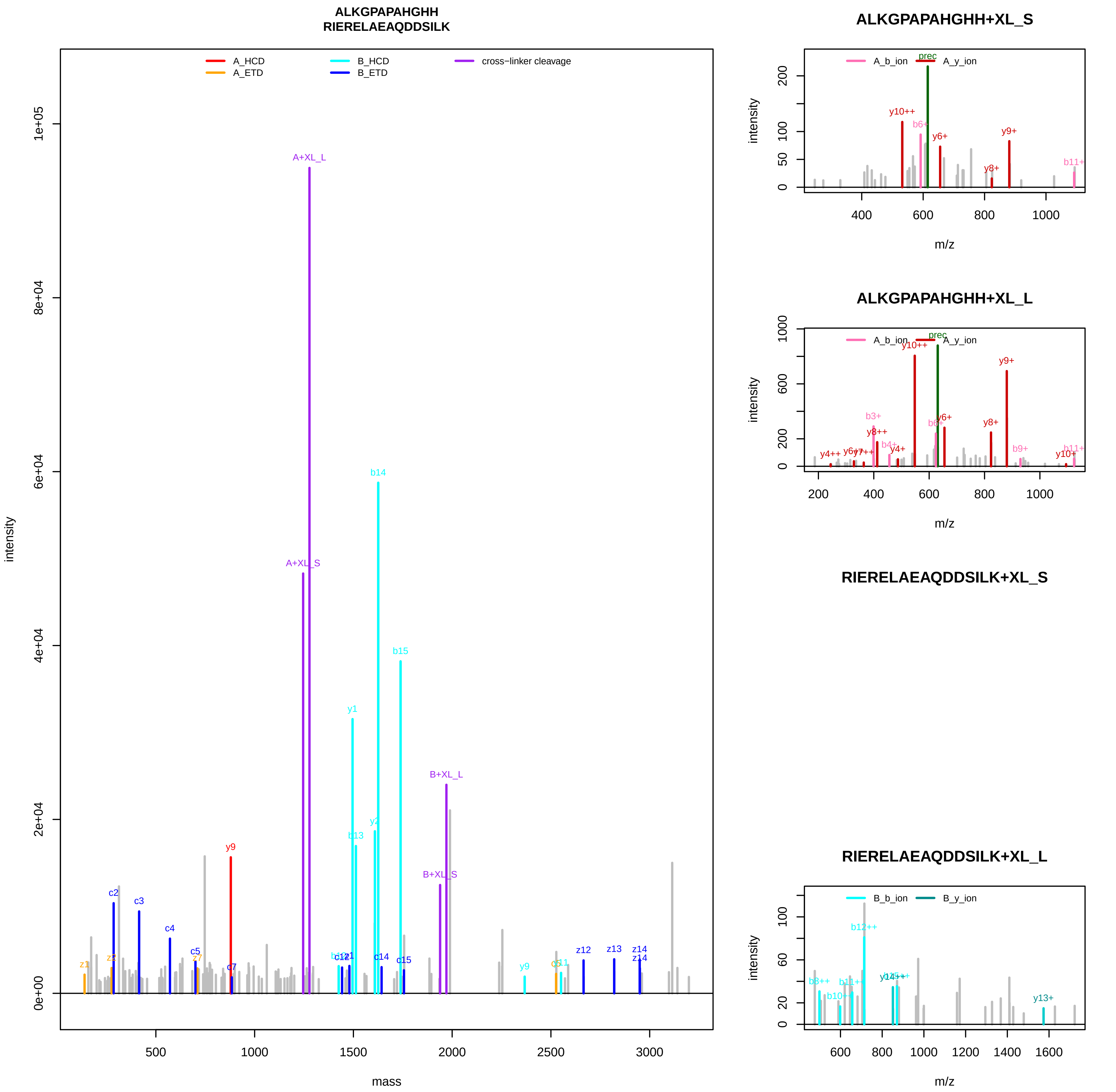
ALKGPAPAHGHH
RIERELAEAQDDSILK
A_HCD
A_ETD
B_HCD
B_ETD
cross−linker cleavage
intensity
mass
A+XL_L
A+XL_S
B+XL_L
B+XL_S
b14
b15
b13
y1
y2
y9
c2
c3
c4
c5
c7
c12
c14
c15
z1
z2
z7
z12
z13
z14
ALKGPAPAHGHH+XL_S
A_b_ion
A_y_ion
prec
y10++
b6+
y6+
y9+
y8+
b11+
m/z
ALKGPAPAHGHH+XL_L
y4++
y8++
y4+
b3+
b4+
b9+
y10+
RIERELAEAQDDSILK+XL_S
RIERELAEAQDDSILK+XL_L
B_b_ion
B_y_ion
b12++
b8++
b10++
b11++
y13+
